# Supplementary material for: FAM210A is essential for cold-induced mitochondrial remodeling in brown adipocytes
Source: Nat Commun. 2023 Oct 10;14:6344. doi: 10.1038/s41467-023-41988-y (PMC10564795; doi:10.1038/s41467-023-41988-y)
Supplement: Supplementary file 1 — Supplementary Information [file 41467_2023_41988_MOESM1_ESM.pdf]

Supplementary information

FAM210A is essential for cold-induced mitochondrial remodeling in brown adipocytes

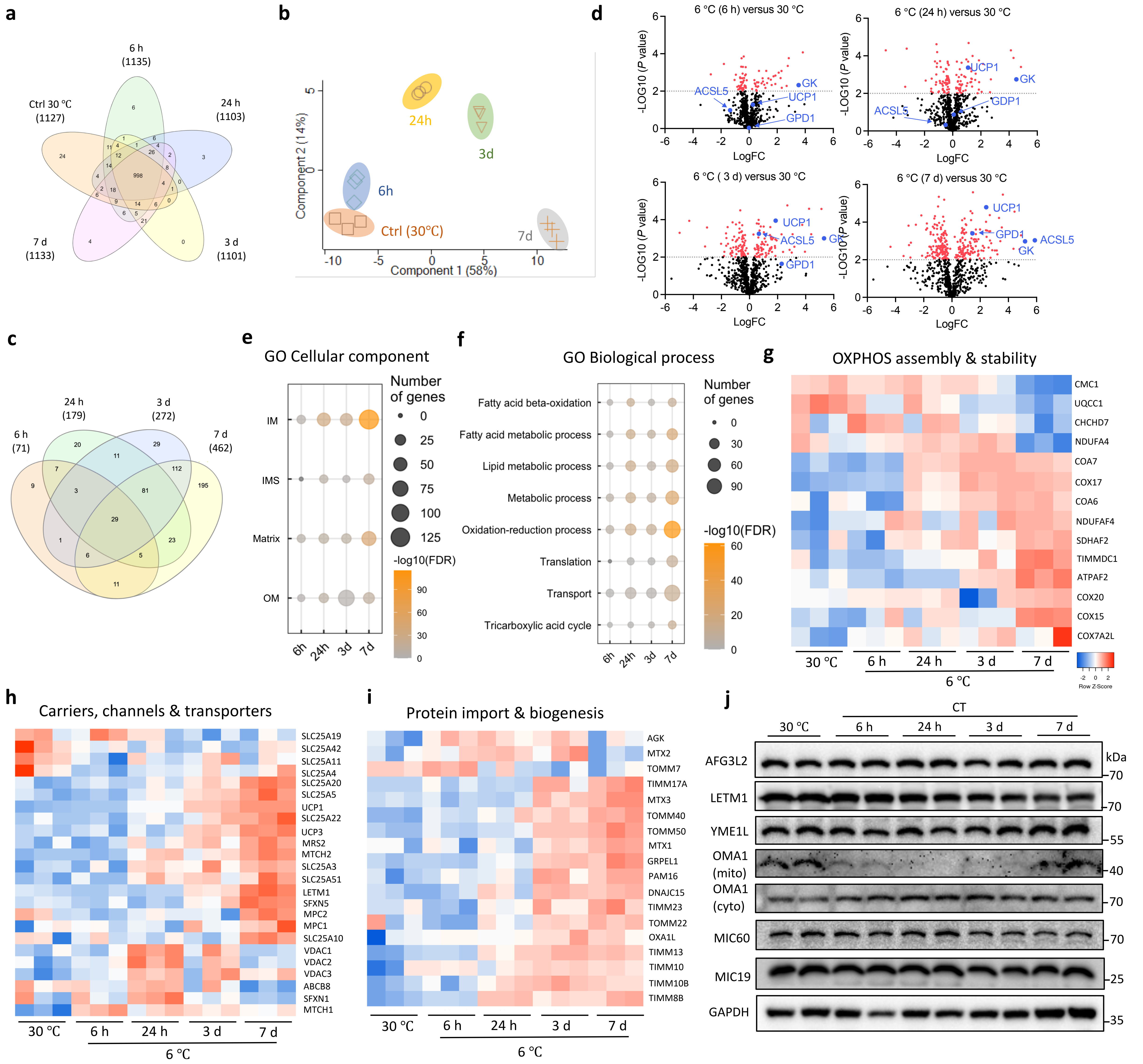

Supplementary Fig. 1 | Changes of BAT mitochondrial proteome during cold exposure. a Venn diagram of all the detected proteins in BAT mitochondrial proteomics during cold exposure. **b** Principal-component analysis (PCA) segregates the five cold treatment groups (n = 3 mice per group). **c** Venn diagram of differentially expressed proteins in BAT mitochondrial proteomics during cold exposure. **d** Volcano plots showing protein abundance differences between different timepoints on cold and thermoneutral environment (30 °C) (n = 3 mice per group; two-tailed unpaired student's t-test; differential expression was determined using a cutoff significance level of $P < 0.01$). Highlighted are BAT activation marker proteins. **e, f** GO-based gene set enrichment for differentially expressed proteins between cold and thermoneutral environment (30 °C). **g-i** Heatmaps of BAT mitochondrial proteins classified in "OXPHOS assembly & stability", "carriers, channels & transporters", and "protein import &biogenesis" at different cold-treatment groups (n = 3 mice per group). Protein classifications were defined based on functional MitoCoP classification. **j** Immunoblotting analysis of proteins related to cristae formation, mitochondrion organization, and mitochondrial fusion (n = 3 mice per group). Source data are provided as a Source Data file.

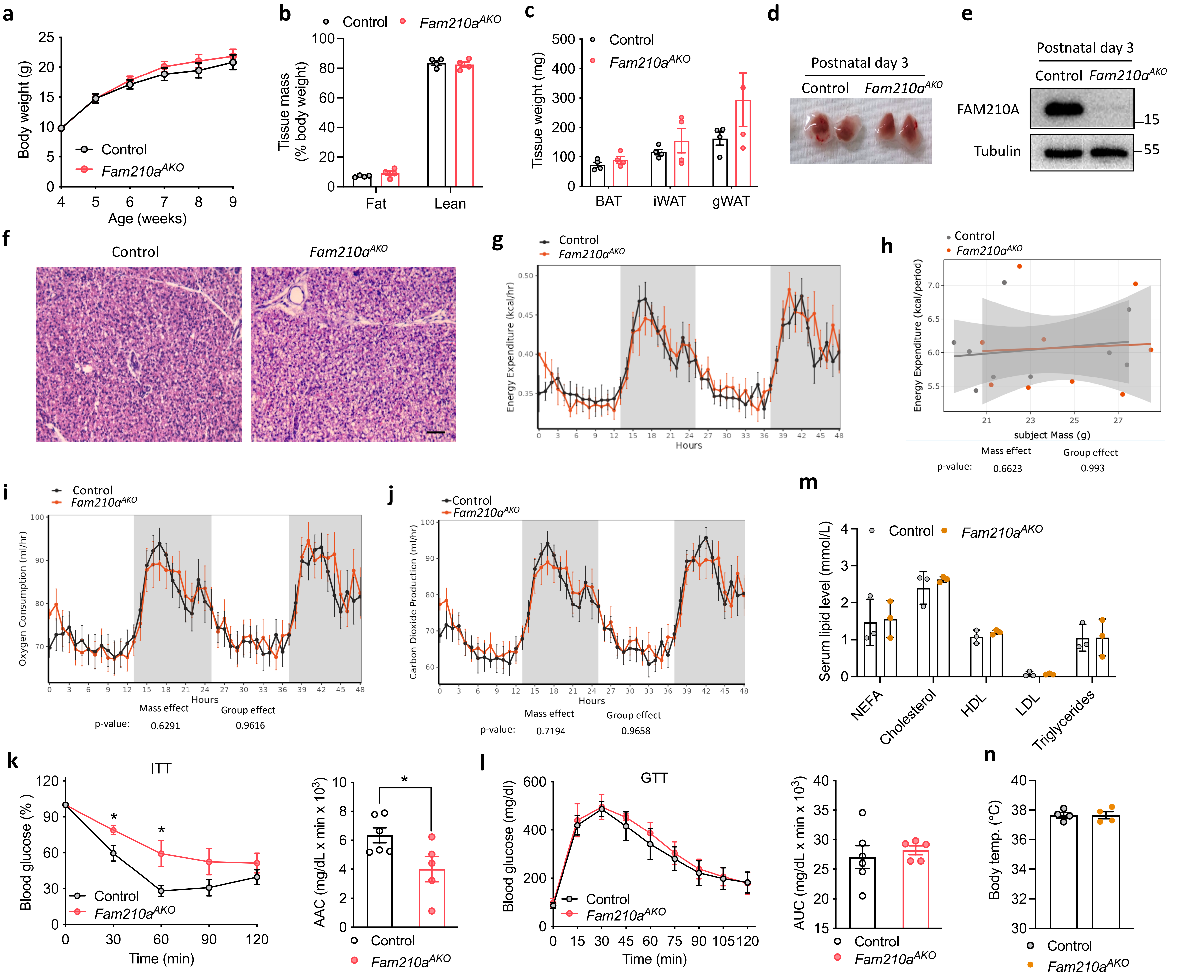

Supplementary Fig. 2 | Loss of *Fam210a* in adipose tissue does not affect systemic metabolism at room temperature. a-c Body weight (**a**), body composition (**b**) and tissue mass (**c**) of control and *Fam210a^{AKO}* mice at RT (n = 10 mice per group for (**a**), n = 7 for (**b**) and (**c**); mean ± s.e.m; two-tailed unpaired student's t-test). **d-f** Representative image of morphology (**d**), immunoblotting analysis (**e**), and H&E staining (**f**) of control and *Fam210a^{AKO}* mice BAT at postnatal day 3 (n = 3 mice per group; scale bar, 50 µm). **g-j** Energy expenditure (**g**), regression-based analysis of energy expenditure (**h**) against body mass, oxygen consumption (**i**), and CO_2 production (**j**) of control and *Fam210a^{AKO}* at RT (n = 9 mice per group; mean ± s.e.m; data were analyzed using CalR-ANCOVA with body mass as a covariate). **k, l** ITT (**k**) and GTT (**l**) of control and *Fam210a^{AKO}* mice at RT (n = 6 mice for control group; n = 5 mice for *Fam210a^{AKO}* group; mean ± s.e.m; two-tailed unpaired student's t-test; *P* = 0.03781, 0.021199, 0.0397). **m** Serum lipid profiles in control and *Fam210a^{AKO}* mice at room temperature (RT) (NEFA, non-esterified fatty acid; HDL, high-density lipoprotein; LDL, low-density lipoprotein; n = 3 mice per group; mean ± s.e.m; two-tailed unpaired student's t-test). **n** Rectal core body temperatures of control and *Fam210a^{AKO}* mice after 7-day cold exposure (n = 4 mice per group; mean ± s.e.m; two-tailed unpaired student's t-test). *$P < 0.05$. Source data are provided as a Source Data file.

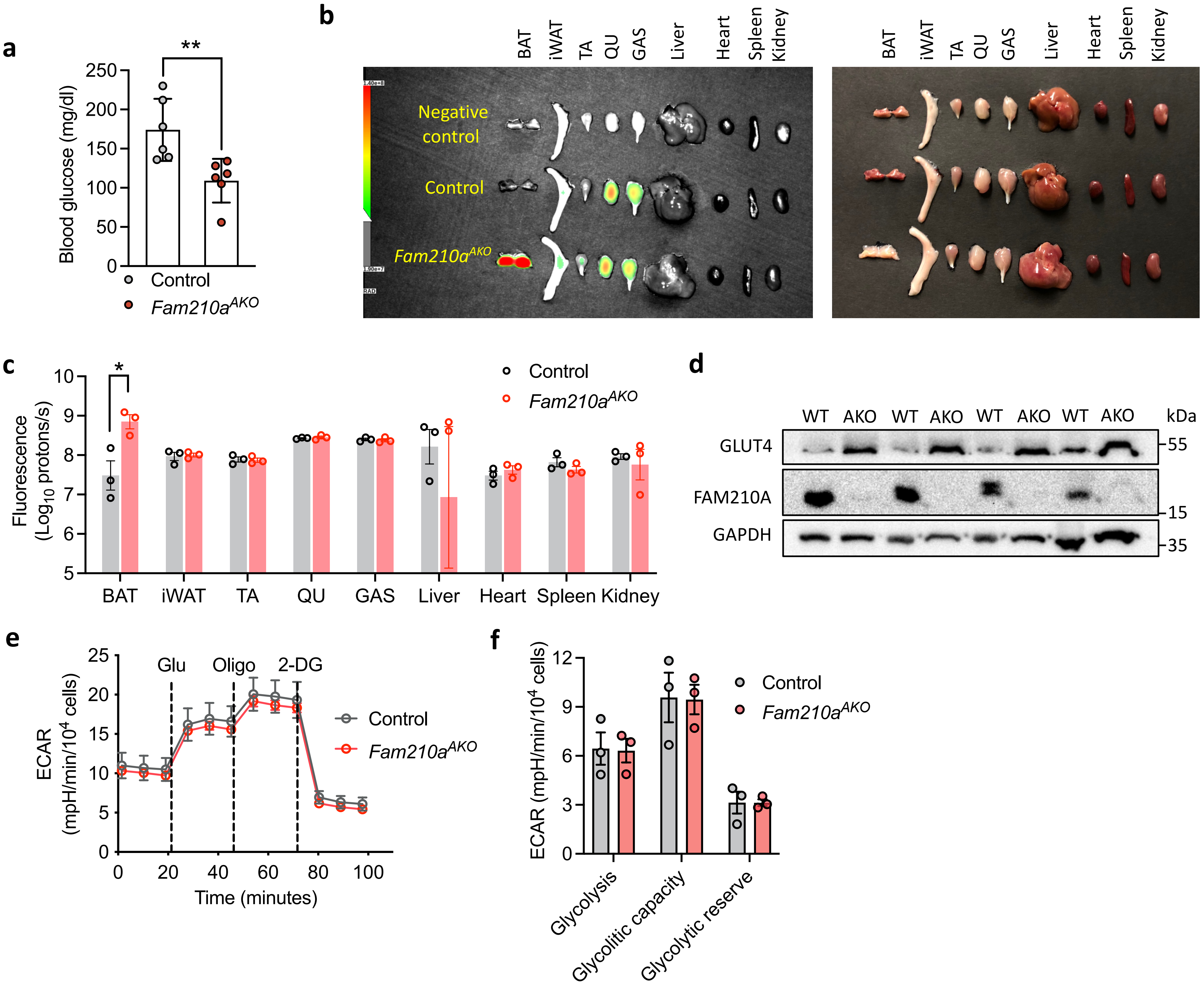

Supplementary Fig. 3 | Loss of *Fam210a* in adipose tissue increases glucose uptake in BAT but does not change glycolysis. a Blood glucose levels of control and *Fam210a*^{AKO} mice at 6 °C for 3 h with fasting (n = 6 mice per group; mean ± s.e.m; two-tailed unpaired student's t-test; *P* = 0.0083). **b** Biodistribution of 2-NBDG in different dissected tissues of control and *Fam210a*^{AKO} mice at 6 °C for 3 h with fasting (TA, tibialis anterior muscle; QU, quadriceps muscle; GAS, gastrocnemius muscle; n = 3 mice per group). **c** Quantitative analysis of fluorescence intensity in (**b**). The fluorescence intensity of each tissue was measured by the optical imaging system and calculated based on the default equation from the system (mean ± s.e.m; two-tailed unpaired student's t-test; *P* = 0.029720). **d** Immunoblotting analysis of GLUT4 of control and *Fam210a*^{AKO} mice (n = 4 mice per group). **e** Extracellular acidification rate (ECAR) of control and *Fam210a*^{AKO} brown adipocytes measured by seahorse XF24 analyzer (Glu, glucose; Oligo, oligomycin; 2-DG, 2-Deoxy-D-glucose; n = 3 biological repeats per group; mean ± s.e.m). **f** Quantification of glycolysis, glycolytic capacity, and glycolytic reserve measured by the ECAR in (**e**) (mean ± s.e.m; two-tailed unpaired student's t-test). **P* < 0.05, ***P* < 0.01. Source data are provided as a Source Data file.

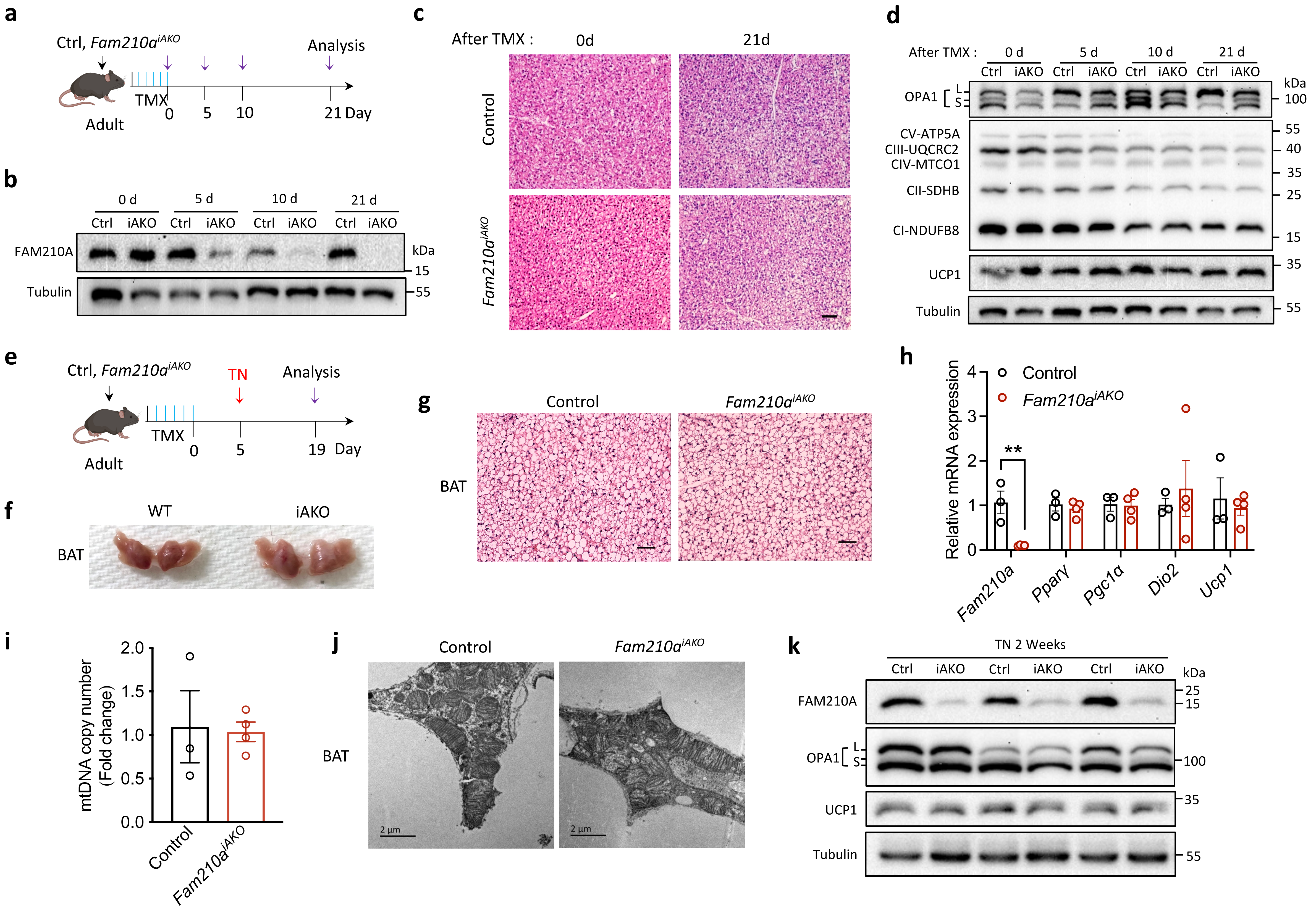

Supplementary Fig. 4 | *Fam210a* is dispensable for BAT mitochondrial maintenance under thermoneutrality. a Schematic showing the timing of tamoxifen (TMX) induction and sampling of control and *Fam210a*^{iAKO} mice (diagram created with BioRender.com). **b** Immunoblotting analysis of FAM210A in BAT from control (Ctrl) and *Fam210a*^{iAKO} (iAKO) mice at different days after TMX (n = 3 mice per group). **c** Representative H&E staining of BAT from control and *Fam210a*^{iAKO} mice at RT (n = 4 mice per group; scale bar: 50 µm). **d** Immunoblotting analysis of mitochondrial proteins in BAT from control (Ctrl) and *Fam210a*^{iAKO} (iAKO) mice at different days after TMX (n = 3 mice per group; scale bar: 50 µm). **e** Schematic showing the timing of tamoxifen (TMX) induction, thermoneutral treatment, and sampling of control and *Fam210a*^{iAKO} mice (diagram created with BioRender.com). **f, g** Representative morphology (**f**) and H&E staining (**g**) of BAT from control and *Fam210a*^{iAKO} mice after 2-week thermoneutral treatment (n = 3 mice per group; scale bars in (**g**): 50 µm). **h** mRNA levels of browning marker genes in BAT from control and *Fam210a*^{iAKO} mice after 2-week thermoneutral treatment (n = 3 mice for control group; n = 4 mice for *Fam210a*^{iAKO} group; mean ± s.e.m; two-tailed unpaired student's t-test; P = 0.006251). **i** Mitochondria copy number in BAT from control and *Fam210a*^{iAKO} mice after 2-week thermoneutral treatment (n = 3 mice for control group; n = 4 mice for *Fam210a*^{iAKO} group; mean ± s.e.m; two-tailed unpaired student's t-test). **j** Representative transmission electron microscopy (TEM) images of BAT from control and *Fam210a*^{iAKO} mice after 2-week thermoneutral treatment (n = 3 mice per group; scale bars, 2 µm). **k** Immunoblotting analysis of BAT from control and *Fam210a*^{iAKO} mice after 2-week thermoneutral treatment (n = 3 mice per group). **P < 0.01. Source data are provided as a Source Data file.

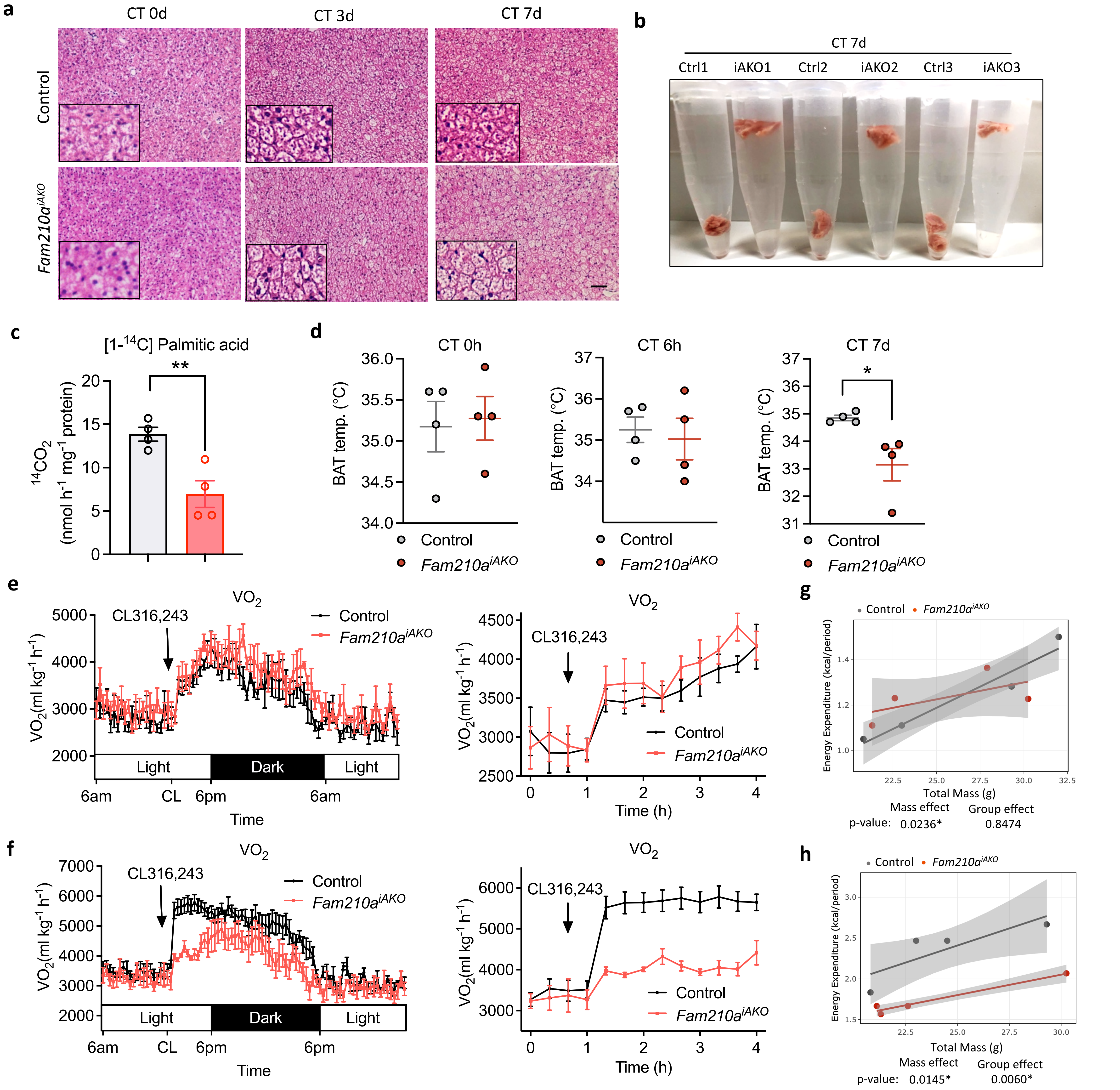

Supplementary Fig. 5 | *Fam210a* is required for cold-induced BAT mitochondrial remodeling. a Representative H&E staining of BAT from control and *Fam210a*^{iAKO} mice after cold exposure for 0 d, 3 d, and 7 d (n = 3 mice per group; scale bar, 50 µm). **b** Representative image showing BATs from control and *Fam210a*^{iAKO} mice fixed in 10% formaldehyde after cold exposure for 7 d (n = 5 mice per group). **c** FAO activity measured using ^{14}C-labeled palmitic acid in BAT from control and *Fam210a*^{iAKO} mice with cold exposure for 7 d (n = 4 mice per group; mean ± s.e.m; two-tailed unpaired student's t-test; $P = 0.0075$). **d** BAT temperature of control and *Fam210a*^{iAKO} mice upon cold exposure for 6 h, 3 d, and 7 d by using temperature microprobe (n = 4 mice per group; mean ± s.e.m; two-tailed unpaired student's t-test; $P = 0.0293$). **e, f** Oxygen consumption of control and *Fam210a*^{iAKO} mice before (**e**) and 7 d after (**f**) cold exposure. **g, h** Regression-based analysis of energy expenditure before (**g**) and after (**h**) cold exposure in (**e, f**) against body mass (n = 4 mice per group; mean ± s.e.m; data were analyzed using CaIR-ANCOVA with energy expenditure as a dependent variable and body mass as a covariate). $^{*}P < 0.05$, $^{**}P < 0.01$. Source data are provided as a Source Data file.

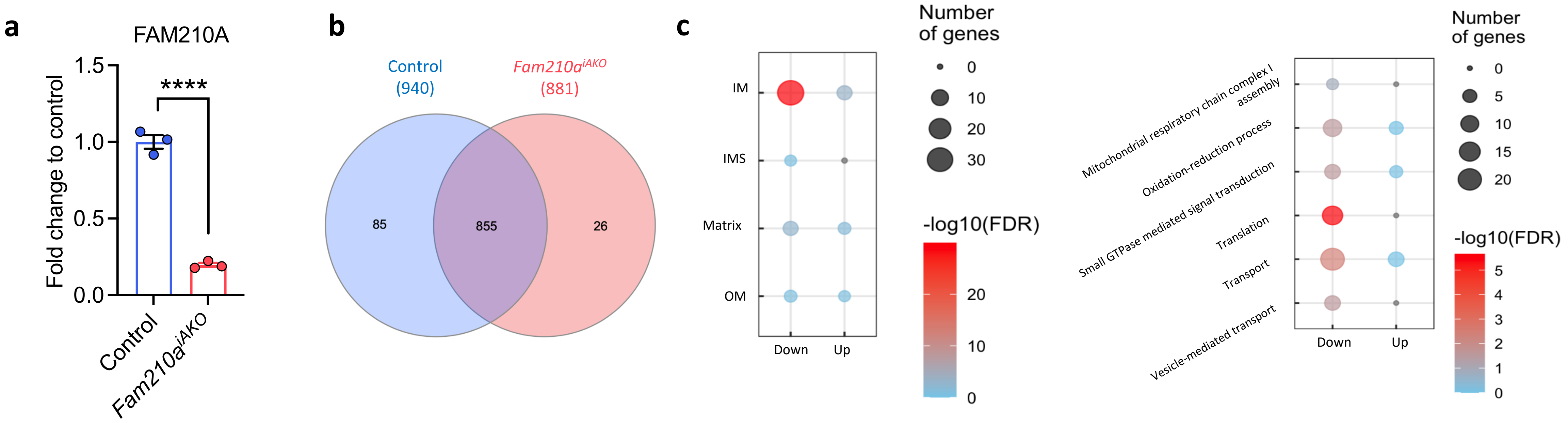

Supplementary Fig. 6 | Changes of BAT mitochondrial proteome in *Fam210a*^{iAKO} mice on cold exposure. a Quantification of FAM210A expression in mitochondrial proteomic data of BAT from control and *Fam210a*^{iAKO} mice upon cold exposure for 3 d (n = 3 mice per group; mean ± s.e.m; two-tailed unpaired student's t-test; P = 0.000063). **b** Venn diagram of proteins detected in BAT mitochondrial proteomics of control and *Fam210a*^{iAKO} mice upon cold exposure for 3 d. **c** GO-based gene set enrichment for differentially expressed proteins between control and *Fam210a*^{iAKO} mice upon cold exposure for 3 d. ****P < 0.0001. Source data are provided as a Source Data file.

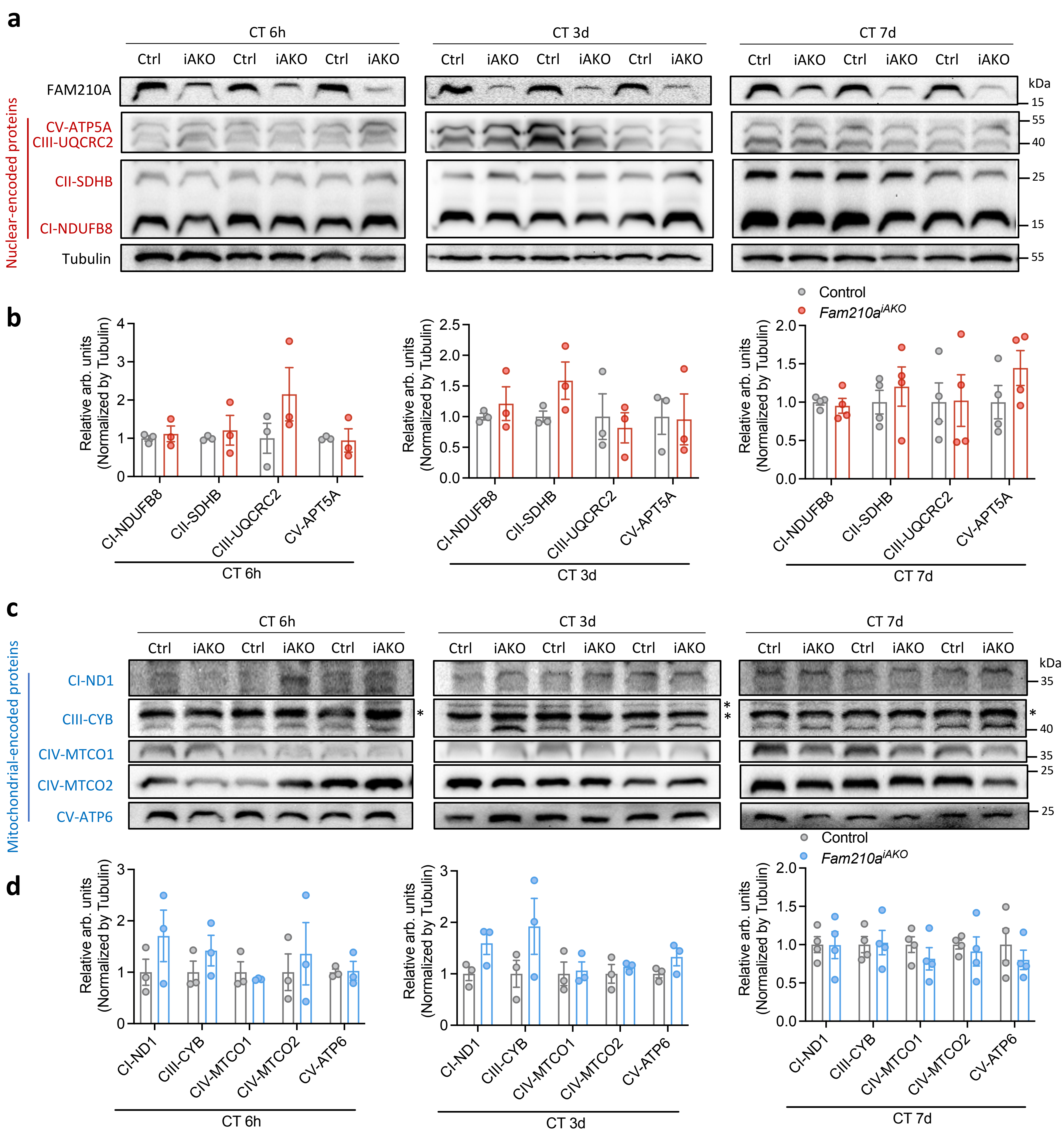

Supplementary Fig. 7 | FAM210A does not directly regulate mitochondrial translation in BAT. a Immunoblotting analysis of nuclear-encoded mitochondrial proteins in BAT from control (Ctrl) and *Fam210a*^{iAKO} (iAKO) mice at different days on cold exposure (n = 3 mice per group for CT 6 h and CT 3 d; n = 4 mice per group for CT 7 d). **b** Quantification of nuclear-encoded proteins in (**a**) (arb. unit, arbitrary unit; mean ± s.e.m; two-tailed paired student's t-test). **c** Immunoblotting analysis of mitochondrial-encoded proteins in BAT from Ctrl and iAKO mice at different days on cold exposure (n = 3 mice per group for CT 6 h and CT 3 d; n = 4 mice per group for CT 7 d; the asterisk indicates non-specific bands). **d** Quantification of nuclear-encoded proteins in (**c**) (mean ± s.e.m; two-tailed paired student's t-test). Source data are provided as a Source Data file.

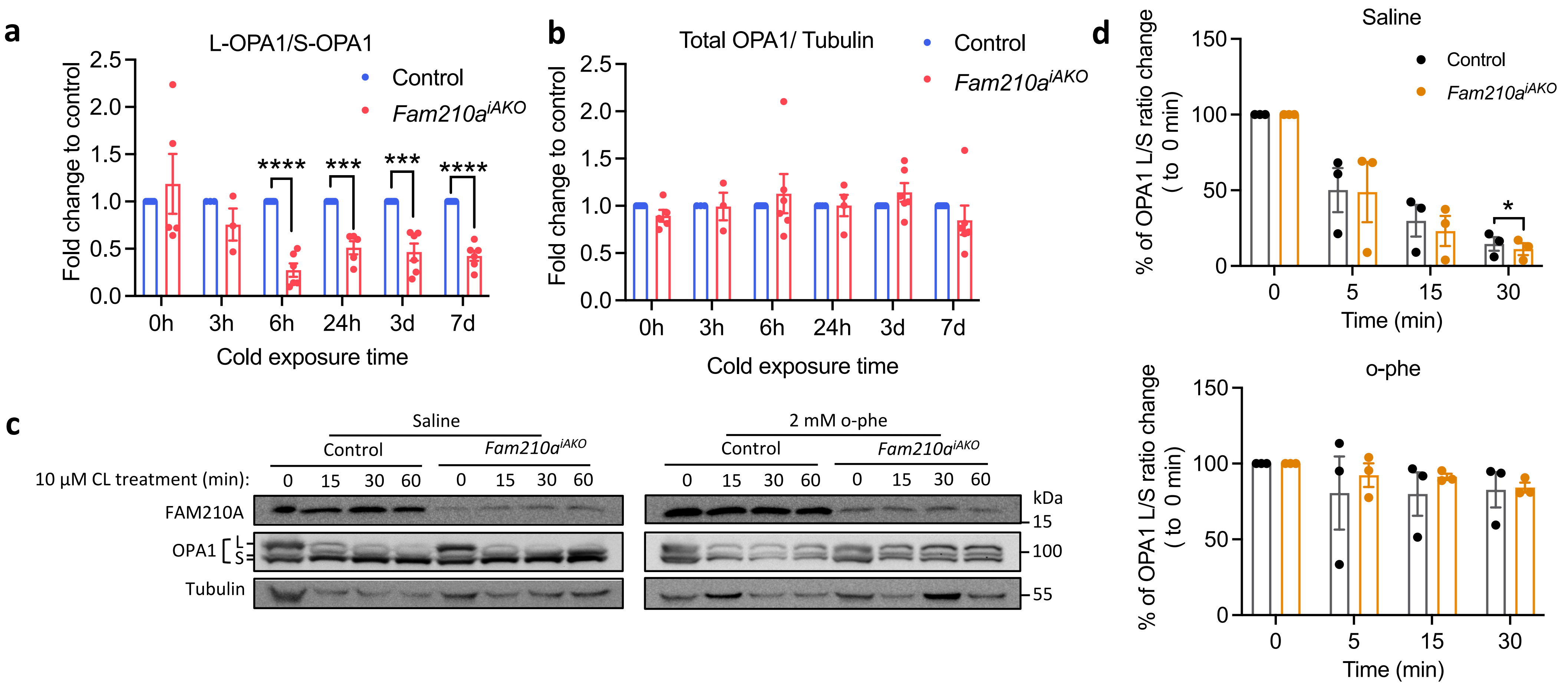

Supplementary Fig. 8 | FAM210A regulates mitochondrial remodeling by OPA1 cleavage. a Quantification of L-OPA1/S-OPA1 fold change to control mice in Fig. 6f (mean ± s.e.m; two-tailed paired student's t-test; P = 0.000153, 0.00226, 0.001959, and 0.000119). **b** Quantification of total OPA1/tubulin in Fig. 6f (mean ± s.e.m; two-tailed paired student's t-test; P = 0.023081). **c** Immunoblotting analysis of OPA1 cleavage of *ex vivo* cultured BAT with or without o-phe treatment (n = 3 independent experiments). **d** Quantification of OPA1 in (**c**) (mean ± s.e.m; two-tailed paired student's t-test). *$P < 0.05$, **$P < 0.01$. Source data are provided as a Source Data file.

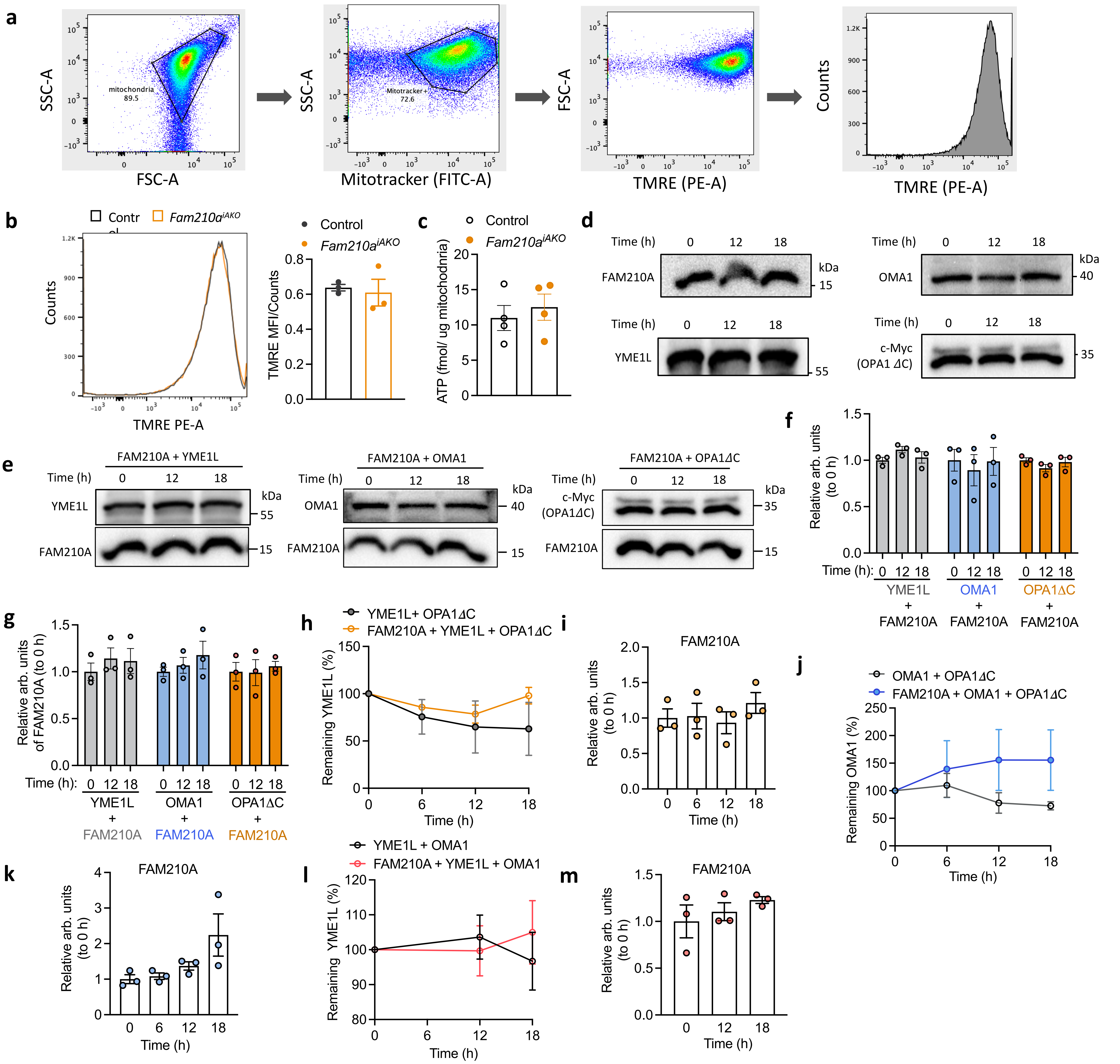

Supplementary Fig. 9 | FAM210A interacts with YME1L and modulates its protease activity toward OMA1 and OPA1 cleavage. a Gating strategies for flow cytometry (SSC-A, Side light scatter; FSC-A, forward light scatter). **b** Measurements of mitochondrial membrane potential by flow cytometry using tetramethylrhodamine ethyl ester (TMRE) with isolated BAT mitochondria (MitoTracker green positive) from control and *Fam210a*^{iAKO} mice 5 d after TMX followed by cold exposure for 24 h. Left: representative TMRE histogram of isolated mitochondria from control and *Fam210a*^{iAKO} mice. Right: quantification of median fluorescence intensity (MFI) to particle counts ratio (n = 3 mice per group; mean ± s.e.m; two-tailed unpaired student's t-test). **c** ATP level of isolated BAT mitochondria from control and *Fam210a*^{iAKO} mice 5 d after TMX followed by cold exposure for 24 h (n = 4 mice per group; mean ± s.e.m; two-tailed unpaired student's t-test). **d** FAM210A, YME1L, OMA1, and OPA1*Δ*C were synthesized by *E. coli* cell-free protein synthesis system and separately incubated for 0 h, 12 h, and 18 h (n = 3 independent experiments). **e** YME1L, OMA1, OPA1*Δ*C, and FAM210A were synthesized by *E. coli* cell-free protein synthesis system, and FAM210A were incubated with YME1L, OMA1, and OPA1*Δ*C, respectively (n = 3 independent experiments). **f, g** Quantification of YME1L, OMA1, OPA1*Δ*C (**f**) and FAM210A protein level (**g**) in (**e**) (n = 3 independent experiments; mean ± s.e.m; two-tailed unpaired student's t-test). **h, i** Quantification of YME1L (**h**) and FAM210A (**i**) protein level in Fig. 7a (n = 3 independent experiments; mean ± s.e.m; two-tailed unpaired student's t-test). **j, k** Quantification of OMA1 (**j**) and FAM210A (**k**) protein level in Fig. 7c (mean ± s.e.m; two-tailed unpaired student's t-test). **l, m** Quantification of YME1L (**l**) and FAM210A (**m**) protein level in Fig. 7g (mean ± s.e.m; two-tailed unpaired student's t-test). Source data are provided as a Source Data file.

Supplementary Table 1. List of antibodies used in this study.

Antibody	Manufacture	Catalog #	Application
Mouse monoclonal anti-FLAG	Sigma	F1804	IF (1:500), IB (1:2000)
Rabbit polyclonal anti-β-Tubulin	Sigma	T2200	IB (1:1000)
Rabbit monoclonal anti-mitofusin 2	Cell signaling	9482	IB (1:1000)
Normal rabbit IgG	Cell signaling	2729	IF (1:300)
Mouse monoclonal anti-OPA1	BD Biosciences	612606	IB (1:1000)
Rabbit polyclonal anti-UCP1	Abcam	ab10983	IHC (1:200), IB (1:1000)
Mouse monoclonal anti-c-Myc	Santa Cruz Biotechnology	sc-40	IF (1:300), IB (1:1000)
Mouse monoclonal anti-GAPDH	Santa Cruz Biotechnology	sc-32233	IB (1:2000)
Rabbit polyclonal anti-Tom20	Santa Cruz Biotechnology	sc-11415	IF (1:300)
Mouse monoclonal anti-Mitofilin	Invitrogen	45-6400	IB (1:1000)
Mouse monoclonal anti-CHCHD3	Invitrogen	MA5-26597	IB (1:1000)
Rabbit polyclonal anti-C18orf19	Invitrogen	PA5-53146	IF (1:300), IB (1:1000)
Mouse monoclonal anti-OxPhos	Invitrogen	45-8099	IB (1:2000)
Rabbit polyclonal anti-AFG3L2	Proteintech	14631-1-AP	IB (1:1000)
Mouse monoclonal anti-GLUT4	Proteintech	66846-1-Ig	IB (1:1000)
Rabbit polyclonal anti-LETM1	Proteintech	1602-1-AP	IB (1:1000)
Rabbit polyclonal anti-OMA1	Proteintech	17116-1-AP	IB (1:1000)
Rabbit polyclonal anti-YME1L	Proteintech	11510-1-AP	IF (1:300), IB (1:1000)
Rabbit polyclonal anti-ATP6	Proteintech	55313-1-AP	IB (1:1000)
Rabbit polyclonal anti-MTCO2	Proteintech	55070-1-AP	IB (1:1000)
Rabbit polyclonal anti-CYTB	Proteintech	55090-1-AP	IB (1:1000)
Rabbit polyclonal anti-ND1	Proteintech	19703-1-AP	IB (1:1000)
Alexa Fluor 488 goat anti-rabbit IgG	Invitrogen	A-11034	IF (1:1000)
Alexa Fluor 647 goat anti-rabbit IgG	Invitrogen	A-21244	IF (1:1000)
CF660C donkey anti-mouse IgG	Biotium	20815	IF (1:500)
HRP AffiniPure goat anti-mouse IgG	Jackson ImmunoResearch	115-035-003	IB (1:10000)
HRP AffiniPure goat anti-rabbit IgG	Jackson ImmunoResearch	111-035-003	IB (1:10000)

Supplementary Table 2. Real-time PCR primer sequences.

**Gene name**	**Sequence (5’ to 3’)**
Cytb: forward	TCATCGACCTCCCCACCCCATC
Cytb: reverse	CGTCTCGAGTGATGTGGGCGATT
B2m: forward	TGGCCATACTACCCTGAATGAGTCC
B2m: reverse	ATGTATTGTGCAATGCTGCTGCTCG
Fam210a: forward	TGACAGCCTACGCCATGTTT
Fam210a: reverse	GGGTTGACATGTAGCCGTGA
Ucp1: forward	AGGCTTCCAGTACCATTAGGT
Ucp1: reverse	CTGAGTGAGGCAAAGCTGATTT
Ppargc1a: forward	TATGGAGTGACATAGAGTGTGCT
Ppargc1a: reverse	CCACTTCAATCCACCCAGAAAG
Dio2: forward	AATTATGCCTCGGAGAAGACCG
Dio2: reverse	GGCAGTTGCCTAGTGAAAGGT
Pparγ: forward	TCGCTGATGCACTGCCTATG
Pparγ: reverse	GAGAGGTCCACAGAGCTGATT
β-actin: forward	GGCTGTATTCCCCTCCATCG
β-actin: reverse	CCAGTTGGTAACAATGCCATGT
