# Supplementary material for: FAM210A is essential for cold-induced mitochondrial remodeling in brown adipocytes
Source: Nat Commun. 2023 Oct 10;14:6344. doi: 10.1038/s41467-023-41988-y (PMC10564795; doi:10.1038/s41467-023-41988-y)
Supplement: Supplementary file 3 — Description of Additional Supplementary Files [file 41467_2023_41988_MOESM3_ESM.pdf]

Description of Additional Supplementary Files

Title: Supplementary Data 1

Description: Differentially expressed BAT mitochondrial proteins of five timepoints during cold exposure corresponding to the Heatmap in Fig.1d.

Title: Supplementary Data 2

Description: Differentially expressed BAT mitochondrial proteins between 30 °C and different timepoints at 6 °C corresponding to the volcano plots in Supplementary Fig. 1d.

Title: Supplementary Data 3

Description: Differentially expressed BAT mitochondrial proteins between control and *Fam210a*^{iAKO} mice on cold for 3 days.
