# Supplementary material for: FAM210A is essential for cold-induced mitochondrial remodeling in brown adipocytes
Source: Nat Commun. 2023 Oct 10;14:6344. doi: 10.1038/s41467-023-41988-y (PMC10564795; doi:10.1038/s41467-023-41988-y)
Supplement: Supplementary file 7 — Reporting Summary [file 41467_2023_41988_MOESM7_ESM.pdf]

nature portfolio

Corresponding author(s): Shihuan Kuang

Last updated by author(s): Aug 29, 2023

Reporting Summary

Nature Portfolio wishes to improve the reproducibility of the work that we publish. This form provides structure for consistency and transparency in reporting. For further information on Nature Portfolio policies, see our Editorial Policies and the Editorial Policy Checklist.

Statistics

For all statistical analyses, confirm that the following items are present in the figure legend, table legend, main text, or Methods section.

n/a	Confirmed	
☐	☒	The exact sample size (n) for each experimental group/condition, given as a discrete number and unit of measurement
☐	☒	A statement on whether measurements were taken from distinct samples or whether the same sample was measured repeatedly
☐	☒	The statistical test(s) used AND whether they are one- or two-sided<br>*Only common tests should be described solely by name; describe more complex techniques in the Methods section.*
☐	☒	A description of all covariates tested
☐	☒	A description of any assumptions or corrections, such as tests of normality and adjustment for multiple comparisons
☐	☒	A full description of the statistical parameters including central tendency (e.g. means) or other basic estimates (e.g. regression coefficient) AND variation (e.g. standard deviation) or associated estimates of uncertainty (e.g. confidence intervals)
☐	☒	For null hypothesis testing, the test statistic (e.g. F, t, r) with confidence intervals, effect sizes, degrees of freedom and P value noted<br>*Give P values as exact values whenever suitable.*
☒	☐	For Bayesian analysis, information on the choice of priors and Markov chain Monte Carlo settings
☒	☐	For hierarchical and complex designs, identification of the appropriate level for tests and full reporting of outcomes
☐	☒	Estimates of effect sizes (e.g. Cohen's d, Pearson's r), indicating how they were calculated

Our web collection on statistics for biologists contains articles on many of the points above.

Software and code

Policy information about availability of computer code

Data collection	Mouse rectal core body temperature was measured by ETI model MicroTherma2 (ETI);<br>Infrared thermography of BAT was aquired by FLIR T440 (Teledyne FLIR);<br>Mouse body composition was measured by EchoMRI 3-in-1 system (Echo Medical Systems),<br>Indirect calorimetry was measured by Oxymax Open Circuit Indirect Calorimeter with Oxymax v4.91 software (Columbus Instruments);<br>Blood glucose level was detected by Roche Accu-Check Active glucometer (Roche Applied Science);<br>Cell oxygen consumption rate and glycolytic rate were measured by Seahorse XFe24 Analyzer (Agilent Technologies);<br>Single-molecule localization data was collected on a custom-built setup on an Olympus IX-73 microscope stand (Olympus America, IX-73) equipped with a 100×/1.35-NA silicone-oil-immersion objective lens (Olympus America, UPLSAPO100XS) and a PIFOC objective positioner (Physik Instrumente, ND72Z2LAQ);<br>Immunoblotting data were detected by Proteinsimple FluorChem R System (Bio-techne);<br>qRT-PCR was conducted using the Roche Light Cycler 480 PCR System (Roche Applied Science);<br>Flow cytometry data were detected by BD LSRFortessa with BD FACSDiva software (BD Biosciences);<br>Microscopy pictures were acquired by Leica DM6000B microscope with MetaMorph V7 software (Molecular Devices) for IF staining images or OMAX ToupView 3.7 (ToupTek) for H&E staining images;<br>In vivo fluorescent images were collected with Ami optical imaging system (Spectral Instruments Imaging);<br>Transmission electron microscopy images were collected with Tecnai T12 transmission electron microscope (Nanoimaging Services);<br>Luminescence intensity was detected by Spark 10M multimode microplate reader (TECAN);<br>Optical density (O.D.) was measure by Nanodrop 2000c (Thermo Fisher);<br>The scintillation was counted by TRI-CARB 1600 liquid scintillation Counter (Packard);

	Protein peptides mass-spectrometry was detected by LTQ-Orbitrap Velos mass spectrometer (Thermo Fisher) coupled to an EASY-nLC 1000 HPLC system (Thermo Fisher).
Data analysis	GraphPad Prism version 8.0 (GraphPad) was used for data analysis;<br>ImageJ 1.52a (National Institutes of Health, USA) was used for immunoblot quantification;<br>Adobe Photoshop CC 19.0 (Adobe System) was used for image processing;<br>MaxQuant v1.6.10.43 was used for proteomics data analysis;<br>Heatmapper was used for proteomic data visualization;<br>FlowJo version 10.8 (BD Biosciences) was used to visualize flow cytometry data;<br>AURA Imaging Software (Spectral Instruments Imaging) was used to anlyze in vivo fluorescent images;<br>RStudio version 2022.12.0+353 was used to visualize proteomics GO analysis data;<br>CalR version 1.3 was used for indirect calorimetry analysis.

For manuscripts utilizing custom algorithms or software that are central to the research but not yet described in published literature, software must be made available to editors and reviewers. We strongly encourage code deposition in a community repository (e.g. GitHub). See the Nature Portfolio guidelines for submitting code & software for further information.

Data

Policy information about availability of data

All manuscripts must include a data availability statement. This statement should provide the following information, where applicable:

- Accession codes, unique identifiers, or web links for publicly available datasets
- A description of any restrictions on data availability
- For clinical datasets or third party data, please ensure that the statement adheres to our policy

The mitochondrial proteomic data generated in this study have been deposited to the ProteomeXchange Consortium via the PRIDE80 partner repository with the dataset identifier PXD044866 (https://www.ebi.ac.uk/pride/archive/projects/PXD044866). All the source data of this study are provided as Source Data file.

Research involving human participants, their data, or biological material

Policy information about studies with human participants or human data. See also policy information about sex, gender (identity/presentation), and sexual orientation and race, ethnicity and racism.

Reporting on sex and gender	N/A
Reporting on race, ethnicity, or other socially relevant groupings	N/A
Population characteristics	N/A
Recruitment	N/A
Ethics oversight	N/A

Note that full information on the approval of the study protocol must also be provided in the manuscript.

Field-specific reporting

Please select the one below that is the best fit for your research. If you are not sure, read the appropriate sections before making your selection.

[x] Life sciences [ ] Behavioural & social sciences [ ] Ecological, evolutionary & environmental sciences

For a reference copy of the document with all sections, see nature.com/documents/nr-reporting-summary-flat.pdf

Life sciences study design

All studies must disclose on these points even when the disclosure is negative.

Sample size	At least three biological replicates per group (all detailed n is indicated in the figure legends) were collected to perform statistical testing within isogenic animal cohorts and ex vivo and in vitro culture experiments. For protein mass-spectrometry, three biological replicates per group were used. No statistical method was used to predetermine sample size. Sample sizes were selected based on the experiment type and the standard practice in the field of genetics and physiology.<br>Relevant reference:<br>1. Sustarsic, Elahu G., et al. "Cardiolipin synthesis in brown and beige fat mitochondria is essential for systemic energy homeostasis." Cell metabolism 28.1 (2018): 159-174.<br>2. Wang, Qiang, et al. "Post-translational control of beige fat biogenesis by PRDM16 stabilization." Nature 609.7925 (2022): 151-158.<br>3. Bi, Pengpeng, et al. "Inhibition of Notch signaling promotes browning of white adipose tissue and ameliorates obesity." Nature medicine 20.8 (2014): 911-918.<br>4. MacVicar, Thomas, et al. "Lipid signalling drives proteolytic rewiring of mitochondria by YME1L." Nature 575.7782 (2019): 361-365.

Data exclusions	Measurement values that were beyond the boundary determined by the interquartile range were considered as outliers and were excluded from statistical analyses.
Replication	All experiments have been done with at least three biological replicates. Replicate experiments were successful and confirmed.
Randomization	For WT mice, the mice were randomly allocated for different times on cold exposure. For control and KO/induced KO mice, we used the mice of same age and sex from the same litter whenever possible, but the paired mice were randomly allocated to experiments including cold, thermoneutral, and CL. Randomization was applied for cell culture, mitochondria isolation, and data collection.
Blinding	After the mice were grouped based on their genotypes, we measured body weight, body temperature, indirect calorimetry, Echo-MRI, GTT, and ITT in a blinded way. Protein mass-spectrometry was done blindly. For other experiments, blinding was not done since the experimental design requires the investigators to know the genotype information, but all results were analyzed in unbiased ways.

Reporting for specific materials, systems and methods

We require information from authors about some types of materials, experimental systems and methods used in many studies. Here, indicate whether each material, system or method listed is relevant to your study. If you are not sure if a list item applies to your research, read the appropriate section before selecting a response.

Materials & experimental systems

n/a	Involved in the study	
☐	☒	Antibodies
☐	☒	Eukaryotic cell lines
☒	☐	Palaeontology and archaeology
☐	☒	Animals and other organisms
☒	☐	Clinical data
☒	☐	Dual use research of concern
☒	☐	Plants

Methods

n/a	Involved in the study	
☒	☐	ChIP-seq
☐	☒	Flow cytometry
☒	☐	MRI-based neuroimaging

Antibodies

Antibodies used

Primary antibodies used in this study:
1. Mouse monoclonal anti-FLAG (Sigma, F1804) was used for IF (1:500) and IB (1:2000);
2. Rabbit polyclonal anti-β-Tubulin (Sigma, T2200) was used for IB (1:1000);
3. Rabbit monoclonal anti-mitofusin 2 (Cell signaling, #9482) was used for IB (1:1000);
4. Rabbit normal IgG (Cell signaling, #2729) was used for IF (1:300).
5. Rabbit polyclonal anti-OPA1 (BD Biosciences, 612606) was used for IB (1:1000);
6. Rabbit polyclonal anti-UCP1 (Abcam, ab10983) was used for IHC (1:200) and IB (1:1000);
7. Mouse monoclonal anti-c-Myc (Santa Cruz Biotechnology, sc-40) was used for IF (1:300) and IB (1:1000);
8. Mouse monoclonal anti-GAPDH (Santa Cruz Biotechnology, sc-32233) was used for IB (1:2000);
9. Rabbit polyclonal anti-Tom20 (Santa Cruz Biotechnology, sc-11415) was used for IF (1:300);
10. Mouse monoclonal anti-Mitofilin (Invitrogen, 45-6400) was used for IB (1:1000);
11. Mouse monoclonal anti-CHCHD3 (Invitrogen, MA5-26597) was used for IB (1:1000);
12. Rabbit polyclonal anti-C18orf19 (Invitrogen, PA5-53146) was used for IF (1:300) and IB (1:1000);
13. Mouse monoclonal anti-OxPhos (Invitrogen, 45-8099) was used for IB (1:2000);
14. Rabbit polyclonal anti-AFG3L2 (Proteintech, 14631-1-AP) was used for IB (1:1000);
15. Mouse monoclonal anti-GLUT4 (Proteintech, 66846-1-lg) was used for IB (1:1000);
16. Rabbit polyclonal anti-LETM1 (Proteintech, 16024-1-AP) was used for IB (1:1000);
17. Rabbit polyclonal anti-OMA1 (Proteintech, 17116-1-AP) was used for IB (1:1000);
18. Rabbit polyclonal anti-YME1L1 (Proteintech, 11510-1-AP) was used for IF (1:300) and IB (1:1000);
19. Rabbit polyclonal anti-ATP6 (Proteintech, 55313-1-AP) was used for IB (1:1000);
20. Rabbit polyclonal anti-MTCO2 (Proteintech, 55070-1-AP) was used for IB (1:1000);
21. Rabbit polyclonal anti-CYTB (Proteintech, 55090-1-AP) was used for IB (1:1000);
22. Rabbit polyclonal anti-ND1 (Proteintech, 19703-1-AP) was used for IB (1:1000).

Secondary antibodies used in this study:
1. Alexa Fluor 488 goat anti-rabbit IgG (Invitrogen, A-11034) was used for IF (1:1000);
2. Alexa Fluor 647 goat anti-rabbit IgG (Invitrogen, A-21244) was used for IF (1:1000);
3. CF660C donkey anti-mouse IgG (Biotium, 20815)was used for IF (1:500);
4. HRP AffiniPure goat anti-mouse IgG (Jackson ImmunoResearch, 115-035-003) was used for IB (1:10000);
5. HRP AffiniPure goat anti-rabbit IgG (Jackson ImmunoResearch, 111-035-003) was used for IB (1:10000).

Validation

All the antibodies used in the study are commercially available and applications have been tested by the manufactures with the validation information that can be found in product sheets or on the manufacturers' websites.

Primary antibodies used in this study:
1. Mouse monoclonal anti-FLAG (Sigma, F1804): https://www.sigmaaldrich.com/US/en/product/sigma/f1804?gclid=CjwKCAjwrranBhAEEiwAzbhNtUOjHD5YK7zPRed94OqGGAKXX9ewNKizycQLl02dj0DrFe8UfcQqaxoCRykQAvD_BwE
2. Rabbit polyclonal anti-β-Tubulin (Sigma, T2200): https://www.sigmaaldrich.com/US/en/product/sigma/t2200
3. Rabbit monoclonal anti-mitofusin 2 (Cell signaling, #9482): https://www.cellsignal.com/products/primary-antibodies/mitofusin-2-d2d10-rabbit-mab/9482?_requestid=292360
4. Rabbit normal IgG (Cell signaling, #2729): https://www.cellsignal.com/products/primary-antibodies/normal-rabbit-igg/2729
5. Rabbit polyclonal anti-OPA1 (BD Biosciences, 612606): https://www.bdbiosciences.com/en-us/products/reagents/microscopy-imaging-reagents/immunofluorescence-reagents/purified-mouse-anti-opa1.612606
6. Rabbit polyclonal anti-UCP1 (Abcam, ab10983): https://www.abcam.com/products/primary-antibodies/ucp1-antibody-ab10983.html
7. Mouse monoclonal anti-c-Myc (Santa Cruz Biotechnology, sc-40): https://www.scbt.com/p/c-myc-antibody-9e10?gclid=CjwKCAjwrranBhAEEiwAzbhNtcvdGkL_70Oprbw3o8dmpdrjM9pfGHlsQujIsald1EB6xvCzt_Fn4RoCtOIQAvD_BwE
8. Mouse monoclonal anti-GAPDH (Santa Cruz Biotechnology, sc-32233): https://www.scbt.com/p/gapdh-antibody-6c5
9. Rabbit polyclonal anti-Tom20 (Santa Cruz Biotechnology, sc-11415): https://datasheets.scbt.com/sc-11415.pdf
10. Mouse monoclonal anti-Mitofilin (Invitrogen, 45-6400): https://www.thermofisher.com/antibody/product/Mitofilin-Antibody-clone-2E4AD5-Monoclonal/45-6400
11. Mouse monoclonal anti-CHCHD3 (Invitrogen, MA5-26597): https://www.thermofisher.com/antibody/product/CHCHD3-Antibody-clone-OTI7G4-Monoclonal/MA5-26597
12. Rabbit polyclonal anti-C18orf19 (Invitrogen, PA5-53146): https://www.thermofisher.com/antibody/product/C18orf19-Antibody-Polyclonal/PA5-53146
13. Mouse monoclonal anti-OxPhos (Invitrogen, 45-8099): https://www.thermofisher.com/antibody/product/OxPhos-Rodent-WB-Antibody-clone-Cocktail-Cocktail/45-8099
14. Rabbit polyclonal anti-AFG3L2 (Proteintech, 14631-1-AP): https://www.ptglab.com/products/AFG3L2-Antibody-14631-1-AP.htm
15. Mouse monoclonal anti-GLUT4 (Proteintech, 66846-1-lg): https://www.ptglab.com/products/GLUT4-Antibody-66846-1-Ig.htm
16. Rabbit polyclonal anti-LETM1 (Proteintech, 16024-1-AP): https://www.ptglab.com/products/LETM1-Antibody-16024-1-AP.htm
17. Rabbit polyclonal anti-OMA1 (Proteintech, 17116-1-AP): https://www.ptglab.com/products/OMA1-Antibody-17116-1-AP.htm
18. Rabbit polyclonal anti-YME1L1 (Proteintech, 11510-1-AP): https://www.ptglab.com/products/YME1L1-Antibody-11510-1-AP.htm
19. Rabbit polyclonal anti-ATP6 (Proteintech, 55313-1-AP): https://www.ptglab.com/products/ATP6-Antibody-55313-1-AP.htm
20. Rabbit polyclonal anti-MTCO2 (Proteintech, 55070-1-AP): https://www.ptglab.com/products/COX2-Antibody-55070-1-AP.htm
21. Rabbit polyclonal anti-CYTB (Proteintech, 55090-1-AP): https://www.ptglab.com/products/CYTB-Antibody-55090-1-AP.htm
22. Rabbit polyclonal anti-ND1 (Proteintech, 19703-1-AP): https://www.ptglab.com/products/ND1-Antibody-19703-1-AP.htm

Secondary antibodies used in this study:
1. Alexa Fluor 488 goat anti-rabbit IgG (Invitrogen, A-11034): https://www.thermofisher.com/antibody/product/Goat-anti-Rabbit-IgG-H-L-Highly-Cross-Adsorbed-Secondary-Antibody-Polyclonal/A-11034
2. Alexa Fluor 647 goat anti-rabbit IgG (Invitrogen, A-21244): https://www.thermofisher.com/antibody/product/Goat-anti-Rabbit-IgG-H-L-Cross-Adsorbed-Secondary-Antibody-Polyclonal/A-21244
3. CF660C donkey anti-mouse IgG (Biotium, 20815): https://biotium.com/product/donkey-anti-mouse-igg-hl-highly-cross-adsorbed-cf-dye-storm/?attribute_pa_conjugation=cf660c
4. HRP AffiniPure goat anti-mouse IgG (Jackson ImmunoResearch, 115-035-003): https://www.jacksonimmuno.com/catalog/products/115-035-003
5. HRP AffiniPure goat anti-rabbit IgG (Jackson ImmunoResearch, 111-035-003): https://www.jacksonimmuno.com/catalog/products/111-035-003

Eukaryotic cell lines

Policy information about cell lines and Sex and Gender in Research

Cell line source(s)	293A cell line (ThermoFisher, R70507), Lenti-X 293T (TaKaRa, 632180), HEK293 (ATCC, CRL-1651), Cos-7 cell line (ATCC, CRL-1651), brown fat cell line (a gift from Dr. Shingo Kajimura).
Authentication	293A cell line (ThermoFisher, R70507), Lenti-X 293T (TaKaRa, 632180), HEK293 (ATCC, CRL-1651), Cos-7 cell line (ATCC, CRL-1651) were purchased from commercial source and not authenticated by the authors. Brown fat cell line was authenticated by UCP1 immunoblotting analysis.
Mycoplasma contamination	All the cell lines were routinely tested for mycoplasma infection and all are tested mycoplasma negative.
Commonly misidentified lines (See ICLAC register)	No commonly misidentified lines were used in this study.

Animals and other research organisms

Policy information about studies involving animals; ARRIVE guidelines recommended for reporting animal research, and Sex and Gender in Research

Laboratory animals	Fam210aflox/flox mouse was generated at Nanjing biomedical research institute of Nanjing University. All other mouse strains were obtained from Jackson Laboratory (Bar Harbor, ME) under the following stock numbers: AdipoqCre (#010803) and AdipoqCreER (#024671). The genotypes of experimental KO and associated control animals are as follows: Fam210aAKO (AdipoqCre+/-;

	Fam210aflox/flox), Fam210aiAKO (AdipoqCreER+/-; Fam210aflox/flox) and control (Fam210aflox/flox). Mice were housed and maintained in the animal facility with free access to water and standard rodent chow food (Teklad Global 19% Protein Extruded Rodent Diet, 2019S), and were housed under 12 h-12 h light-dark cycle, at 22°C, and 45% humidity on average. For all animal-based experiments, at least three pairs of gender-matched littermates at age of 2- to 5-month-old were used for all experiments. Cells or tissues were isolated or collected from the above mouse strains.
Wild animals	The study did not involve any wild animals.
Reporting on sex	Male or female mice were used and always gender matched for each specific experiment.
Field-collected samples	The study did not involve any samples collected from the field.
Ethics oversight	This research complies with all relevant ethical regulations. All procedures involving animals were done in compliance with National Institutes of Health and Institutional guidelines with approval by the Purdue Animal Care and Use Committee.

Note that full information on the approval of the study protocol must also be provided in the manuscript.

Flow Cytometry

Plots

Confirm that:

- [x] The axis labels state the marker and fluorochrome used (e.g. CD4-FITC).
- [x] The axis scales are clearly visible. Include numbers along axes only for bottom left plot of group (a 'group' is an analysis of identical markers).
- [x] All plots are contour plots with outliers or pseudocolor plots.
- [x] A numerical value for number of cells or percentage (with statistics) is provided.

Methodology

Sample preparation	Isolated mitochondria were stained with MitoTracker green (Invitrogen, cat#M7514; 1:1000) and tetramethylrhodamine ethyl ester (TMRE; Cayman chemical, cat#21426; 1:1000) for 10 min on ice before analysis.
Instrument	Samples were analyzed using BD LSRFortessa cell analyzer.
Software	Raw data were collected with BD FACSDiva software and FlowJo version 10.8, and analyzed with GraphPad Prism 8.0.
Cell population abundance	Isolated mitochondria after staining were analyzed on a BD LSRFortessa with BD FACSDiva software. MitoTracker green was detected in the FITC channel and TMRE in the PE-channel. Mitotracker positive mitochondria were determined to be > 70%.
Gating strategy	Isolated mitochondria were gated on FSC/SSC (mean FSC-A: ~10,000/mean SSC-A: ~10,000). Mitochondria were further gated on SSC-A vs. FITC-A for Mitotracker Green positive (FITC intensity > ~1000) particles. Pure mitochondria were determined by gating on a negative population (no Mitotracker Green staining).

- [x] Tick this box to confirm that a figure exemplifying the gating strategy is provided in the Supplementary Information.
